# The Impact of Self-Reported Hearing Difficulties on Memory Collaboration in Older Adults

**DOI:** 10.3389/fnins.2019.00870

**Published:** 2019-08-27

**Authors:** Amanda J. Barnier, Celia B. Harris, Thomas Morris, Paul Strutt, Greg Savage

**Affiliations:** ^1^Australian Research Council Centre of Excellence in Cognition and Its Disorders, Macquarie University, Sydney, NSW, Australia; ^2^Department of Cognitive Science, Macquarie University, Sydney, NSW, Australia; ^3^Dementia Centre, HammondCare, Greenwich, NSW, Australia; ^4^Department of Psychology, Macquarie University, Sydney, NSW, Australia

**Keywords:** memory, aging, collaborative recall, conversation, transactive memory, distributed cognition, hearing loss, presbycusis

## Abstract

Cognitive scientists and philosophers recently have highlighted the value of thinking about people at risk of or living with dementia as intertwined parts of broader cognitive systems that involve their spouse, family, friends, or carers. By this view, we rely on people and things around us to “scaffold” mental processes such as memory. In the current study, we identified 39 long-married, older adult couples who are part of the Australian Imaging Biomarkers and Lifestyle (AIBL) Study of Ageing; all were cognitively healthy but half were subjective memory complainers. During two visits to their homes 1 week apart, we assessed husbands’ and wives’ cognitive performance across a range of everyday memory tasks working alone (Week 1) versus together (Week 2), including a Friends Task where they provided first and last names of their friends and acquaintances. As reported elsewhere, elderly couples recalled many more friends’ names working together compared to alone. Couples who remembered successfully together used well-developed, rich, sensitive, and dynamic communication strategies to boost each other’s recall. However, if one or both spouses self-reported mild-to-moderate or severe hearing difficulties (56% of husbands, 31% of wives), couples received less benefit from collaboration. Our findings imply that hearing loss may disrupt collaborative support structures that couples (and other intimate communicative partners) hone over decades together. We discuss the possibility that, cut off from the social world that scaffolds them, hearing loss may place older adults at greater risk of cognitive decline and dementia.

## Introduction

Across a lifetime in intimate relationships involving joint memory and action, people form expert “remembering systems” that may have (much) later cognitive payoffs (Harris et al., 2011, 2014a; Barnier et al., 2018a). Intimate partners, family members, and friends form complex, distributed “transactive memory systems” (Wegner et al., 1985; Wegner, 1987) that allow them to accomplish more together than when they work alone (Barnier et al., 2008, 2018b; Harris et al., 2014b). However, this “collaborative benefit” is not observed in all groups. In fact, most studies of memory collaboration involving either younger or older pairs of strangers demonstrate “collaborative inhibition,” where individuals recalling separately outperform groups on tests of memory recall (see Basden et al., 1997; Weldon and Bellinger, 1997; Harris et al., 2008; Meade and Roediger, 2009; Rajaram, 2011; Marion and Thorley, 2016).

However, studies of collaborative memory in stranger dyads do not capture well the high levels of shared knowledge developed over the course of a lifetime of shared experiences, and rarely consider the degree to which collaborative benefits may be more (or less) related to the type of information (personal or non-personal) to be remembered (Barnier et al., 2013; Dixon, 2013). Recent work conducted by our group has sought to extend the standard collaborative recall paradigm to older, long-married couples who share a lifetime of remembering together (e.g., Harris et al., 2011, 2017; Barnier et al., 2018a); the kinds of groups that might be expected to develop transactive memory systems (Wegner, 1987; Barnier et al., 2018b). We also have investigated the degree to which tasks that involve recalling personal shared knowledge, rather than non-personal recall tasks, might lead to different patterns of collaborative success and failure. Results indicate that older, long-married couples benefit from collaboration on memory recall tasks, particularly those that require recall of personal shared knowledge (e.g., names of friends and acquaintances) (Barnier et al., 2018a). However, not all couples benefit from collaboration in the same way, despite sharing much of their adult lives together. Even among long-married couples, we find substantial individual differences in the extent to which they collaborate effectively. To gain a better understanding of why this is the case, we and other researchers have transcribed, coded, and analyzed collaborating couples’ (and other dyads’) conversations to identify characteristics of their communication that lead to the greatest benefits during collaborative recall (e.g., Johansson et al., 2005; Vredeveldt et al., 2016; Harris et al., 2018). In other words, it is not merely the length of a relationship that predicts collaborative success, but the ability to effectively communicate with one another (see also Harris et al., 2014b).

Given that our work so far has shown that collaboration can benefit recall performance and that communication is central to this success, it is important to consider factors that may disrupt communication. The ability to hear connects us to the world and the people around us and is fundamental to everyday cognitive and emotional health and well-being (Beechey et al., 2018). Age-related hearing loss is highly prevalent in older adults; the World Health Organization estimates that 1 in 3 people over 65 years of age are affected by disabling hearing loss (Chia et al., 2007; World Health Organization, 2019). Hearing loss also is a significant risk factor for dementia (Lin et al., 2011; Livingston et al., 2017; Jayakody et al., 2018), suggesting a possible association between hearing and memory function. Given this association, and the body of work demonstrating benefits of collaboration for memory recall in older adults, it is important to investigate whether hearing loss disrupts access to the benefits of shared remembering. If family and friends scaffold our cognition *via* joint collaborative remembering, and we in turn help to scaffold them (Harris et al., 2014b; Barnier et al., 2018a, b), we may lose the potential cognitive and memory benefits associated with collaboration if we are less able to hear and communicate with the people around us.

In the current study, long-married older adults completed a self-report questionnaire about the extent to which they experience social and emotional difficulties related to hearing loss, and completed a series of memory tasks individually and with their spouse. We expected that if at least one member of a couple reported everyday hearing difficulties, it would disrupt their ability to remember together and reduce collaborative memory benefits. Further, we expected an association between severity of hearing loss within couples and collaborative benefits, whereby couples reporting the greatest level of hearing difficulty would benefit least from collaboration with their spouse.

## Method

### Participants

Participants were 78 men and women (39 men, 39 women) aged 68–90 years old (*M* = 74.74, *SD* = 5.10). These individuals formed 39 male–female couples, who had been married for 13–65 years (*M* = 49.46, *SD* = 8.78). Participants were a subset of those from the Australian Imaging Biomarkers and Lifestyle Study of Ageing (AIBL; Ellis et al., 2009) and had been classified as cognitively healthy based on their most recent AIBL assessment, as well as their performance on the Mini-Mental State Examination (MMSE; Folstein et al., 1975) on the day of our testing (see also Barnier et al., 2018a; Harris et al., 2018). However, half of our sample were classified as subjective memory complainers because, despite being cognitively healthy according to objective measures, they answered yes to the question “Do you have difficulties with your memory?” at their last AIBL assessment.

The AIBL Study was established in 2006 with 1,112 individuals recruited during the baseline phase. They underwent a screening interview, cognitive and mood assessments, and blood-based biomarker analyses, and completed health and lifestyle questionnaires. Approximately a quarter of the sample underwent brain imaging, including magnetic resonance imaging (MRI) and Pittsburgh compound B–positron emission tomography (PiB-PET). A clinical review panel considered all medical, psychiatric, neuropsychological, and health data and classified 768 as healthy controls, 133 as having mild cognitive impairment (MCI; Petersen et al., 1999; Winblad et al., 2004), and 211 as having Alzheimer’s disease (McKhann et al., 1984). Follow-up assessments of participants have occurred approximately every 18 months, with Wave 4 testing occurring prior to our data collection, and 54 months following initial baseline testing. Further details of the study and baseline characteristics are reported in Ellis et al. (2009). For the current study, we identified 94 individuals who were being tracked as healthy controls within the AIBL sample, and who happened to be married to another AIBL participant (i.e., the whole AIBL sample contained 47 married couples). We first contacted couples *via* a letter inviting them to participate and confirmed their interest by telephone. Seventy-eight individuals (39 couples) were interested and available to participate, and these were our participants for the current study.

### Measures and Procedure

The Macquarie University Human Research Ethics Committee provided ethical approval for this research and the AIBL Management Committee approved access to their participants and the study design. Prior to the experimental sessions, all participants received and completed the Hearing Handicap Inventory for the Elderly – Screening Version (HHIE-S; Ventry and Weinstein, 1983). The HHIE-S is a 10-item questionnaire measuring perceived social and emotional impacts of hearing loss using a self-report format. Responses are provided on a three-point scale “Yes” (4 points), “Sometimes” (2 points), “No” (0 points). Scores range from 0 to 40; scores of 0–8 typically indicate no (self-reported) hearing difficulties; scores of 10–24 typically indicate mild-to-moderate hearing difficulties; and scores of 26–40 typically indicate severe hearing difficulties.

On the day of testing in Week 1, participants also completed the MMSE (Folstein et al., 1975) and the Geriatric Depression Scale – Short Form (GDS-SF; Yesavage and Sheikh, 1986). The MMSE is a brief screen of general cognitive ability including items that assess orientation, registration, attention, recall, language, and visuospatial ability. The MMSE is scored out of 30; scores of 24 or above typically indicate healthy cognition. The GDS-SF is a brief measure of depressive symptoms including “yes” or “no” questions regarding how the participant felt over the last week. The GDS-SF is scored out of 15; scores of 6 or above typically indicate depressive symptoms requiring further investigation. Our participants had a mean MMSE score of 28.87 (*SD* = 1.48) and a mean GDS-SF score of 1.08 (*SD* = 1.38).

Individual and collaborative sessions were conducted in participants’ homes, 1 week apart. Week 1 involved an individual recall session, while Week 2 involved a collaborative recall session. During each of the two sessions, participants completed a range of memory tasks that varied by type and degree of personal significance of the information recalled. Overall performance across these tasks is reported elsewhere (Barnier et al., 2018a). We focus in this paper on a “Mutual Friends” recall task, where participants were required to recall the names of as many mutual friends and acquaintances as possible in 2 min. In Week 1, two experimenters administered the recall tasks individually but simultaneously in separate rooms of the couples’ home. In Week 2, the experimenters returned to the couples’ home and tested participants together on the same recall tasks in a collaborative recall session. Participants were not reimbursed for their involvement in our study; however, morning or afternoon tea was provided by the experimenters. We focus in particular on the impact of hearing loss on couples’ individual and collaborative performance.

## Results

### Influences of Collaboration on Recall

In the collaborative recall paradigm, the impact of recalling together is indexed by comparing collaborative output with the pooled, non-redundant output of individuals recalling alone. As reported in Barnier et al. (2018a), a *t*-test revealed that couples recalled significantly more names of mutual friends together in Week 2 (*M_*recall*_* = 47.64, *SD* = 17.99) compared to their pooled individual or “nominal” recall in Week 1 (*M_*recall*_* = 30.85, *SD* = 12.34); a paired-samples *t*-test comparing these scores was significant, *t*(38) = 10.02, *p* < 0.001. Therefore, on average, couples showed collaborative facilitation on the Mutual Friends task, recalling 16.79 more names (*SD* = 10.46) when they remembered together compared to separately. However, underneath this group level performance, there was considerable variability in the degree of collaborative benefit achieved by each couple. We indexed individual differences in the outcomes of collaboration by assigning each couple a “collaborative benefit” score, which was the difference between the number of names couples recalled during collaboration and the combined number of names recalled by husbands and wives during their individual recall. While no couples showed inhibited recall during collaboration compared to their nominal performance, collaborative benefit scores ranged from 0 to 38 extra names. Whereas 25% of couples gained six or fewer extra names when they collaborated, 25% gained 24 or more extra names. In other words, whereas some couples collaborated very successfully, others gained little or no benefit in terms of performance when collaborating with their spouse. Do hearing difficulties help to explain why?

### Impact of Hearing Difficulties on Individual Memory

On the HHIE-S, individual scores ranged from 0 to 32 (out of 40). Table 1 presents the number of participants who reported no hearing difficulties (0–8), mild-to-moderate hearing difficulties (10–24), and severe hearing difficulties (26–40) as well as mean HHIE-S scores and other demographic information. Analysis revealed some differences in the characteristics of participants across these three hearing classifications (see Table 1). A chi-square analysis of frequencies indicated a trend toward gender differences, such that more men than women tended to appear in the two hearing loss categories, χ^2^(2,78) = 5.37, *p* = 0.068. The increased hearing difficulties reported by men may reflect a greater willingness by men to report or the fact that, within couples, husbands (*M*_*age*_ = 76.15, *SD* = 5.44) were slightly, but significantly, older than wives (*M*_*age*_ = 73.33, *SD* = 4.37), *t*(38) = 5.08, *p* < 0.001. Indeed, we found significant age differences across participants in the different hearing classifications. A one-way analysis of variance (ANOVA) of hearing group on age was significant, *F*(2, 77) = 8.65, *p* < 0.001. Follow-up comparisons (with a Bonferroni adjustment on reported *p*-values) suggested that each of the three hearing classifications differed from the others in age either marginally or significantly, with a pattern that hearing difficulties increased with age, all *p*s < 0.073 (see Table 1).

**TABLE 1:** Frequency of self-reported hearing difficulties, with mean HHIE-S scores, demographics, and individual recall scores.

	**No Hearing Difficulties**	**Mild–Moderate Hearing Difficulties**	**Severe Hearing Difficulties**
Number of participants	44	27	7
Hearing difficulties (HHIE-S)	3.41 (2.78)	14.07 (3.70)	28.57 (2.23)
Sex (% female)	61.4	37.0	28.6
Age	73.14 (4.37)	75.89 (5.12)	80.43 (4.58)
MMSE	29.00 (1.33)	28.85 (1.73)	28.14 (1.35)
Subjective memory complainers (%)	45.5	59.3	57.1
Depression (GDS)	0.84 (1.28)	1.19 (1.21)	2.14 (2.19)
Years of education	14.37 (4.23)	14.85 (4.24)	13.5 (2.88)
Individual recall	19.30 (10.08)	19.26 (7.34)	14.29 (10.19)
*Range*	*3–46*	*4–33*	*0–25*

*Unless otherwise stated, values are means with standard deviations in parentheses.*

Participants across hearing classifications did not differ in cognitive measures. A one-way ANOVA of hearing group on MMSE scores was not significant, *F*(2, 77) = 1.02, *p* = 0.367, and neither was a chi-square analysis of the frequencies of subjective memory complainers in each group, χ^2^(2,78) = 1.38, *p* = 0.501. There was a marginal main effect suggesting increased depression symptomatology with increased severity of hearing difficulties, *F*(2, 77) = 2.94, *p* = 0.059, but across classifications, participants’ average GDS scores were well below the clinical cutoff of 6 (see Table 1).

On the Week 1 individual memory test, HHIE-S scores were not correlated with the number of names participants recalled, *r* = -0.086, *p* = 0.452. Instead, across our three hearing groups, a one-way ANOVA showed that participants recalled a similar number of names, *F*(2, 77) = 0.88, *p* = 0.418 (see Table 1). Self-reported hearing difficulties did not influence Week 1 individual memory performance on the Mutual Friends task when participants recalled alone in the presence of an experimenter.

### Impact of Hearing Difficulties on Collaborative Memory

In Week 2, we explicitly instructed couples to “work together” to recall as many names as possible. Since we analyzed collaborative recall at the couple level, we classified couples into three groups to mirror our individual hearing classifications: (1) no hearing difficulties reported (*n* = 12 couples; 30.7%), (2) at least one spouse with mild hearing difficulties (*n* = 20 couples; 51.3%); and (3) at least one spouse with severe hearing difficulties (*n* = 7 couples; 17.9%). We conducted a one-way ANOVA to compare collaborative benefit scores across these three groups. This analysis yielded a significant main effect of group, *F*(2, 38) = 3.57, *p* = 0.039, and a significant linear function, *F*(2, 38) = 7.14, *p* = 0.011. Follow up comparisons (with a Bonferroni adjustment on reported *p*-values) indicated that couples in which at least one member reported severe hearing difficulties collaborated far less successfully (*M_*benefit*_* = 8.86, *SD* = 9.21) than couples in which neither member reported hearing difficulties (*M_*benefit*_* = 21.33, *SD* = 11.57), *p* = 0.034. The benefit scores for couples in which at least one member reported mild-to-moderate difficulties (*M_*benefit*_* = 16.85, *SD* = 8.86) fell in the middle and was not significantly different from the other two groups, *p*s > 0.21. The linear function suggests that collaborative benefit decreased as hearing difficulties within the couples increased.

To further examine this linear relationship between hearing difficulties and collaborative benefit within each couple, we added husbands’ and wives’ individual HHIE-S scores to create a measure of couple-level hearing difficulties. Higher additive scores indicate more reported hearing problems by the couples (possible range: 0–80). Couples’ additive scores ranged from 4 to 56 (*M*_*couple*_ = 18.72, *SD* = 11.69). This range suggests that it was relatively uncommon for both partners to report very high levels of hearing difficulties. Indeed, there were no cases in which both spouses reported severe hearing difficulties, only 3 (7.7%) cases in which one spouse reported significant difficulties and the other reported mild-to-moderate difficulties, and only 4 (10.3%) cases in which both spouses reported mild-to-moderate hearing difficulties. Instead, in 20 out of 39 cases (51.3%), one of the partners reported (mild-to-moderate or severe) hearing difficulties and the other reported no difficulties. In other words, many couples showed asymmetrical profiles with one spouse struggling to hear more than the other. Consistent with the linear relationship described above, couples’ combined hearing difficulty scores correlated negatively with their collaborative benefit scores, *r* = -0.34, *p* = 0.033 (two-tailed). Overall, our results suggest that greater hearing difficulties within couples’ “systems” reduced the success of their memory collaboration.

## Discussion

In this study, we aimed to identify whether self-reported hearing difficulties reduced the benefits of collaborative recall in older, long-married couples. Because the benefits of collaboration are driven by effective communication (Harris et al., 2011, 2018; see Vredeveldt et al., 2016, for similar findings in a forensic context), we expected that hearing difficulties might reduce couples’ ability to communicate and collaborate successfully. Self-reported hearing difficulties were not related to individual recall performance, and this may reflect the fact that recalling names of friends and acquaintances alone in Week 1 did not depend on discussion with anyone else. However, during collaboration in Week 2, couples benefited less from the opportunity to recall with their spouse when at least one member of the couple reported hearing difficulties. Moreover, the impact of these difficulties appeared additive, with couples’ combined scores associated with less successful collaboration. Therefore, our results, while exploratory, suggest that hearing difficulties reduce the benefits of remembering with a close collaborative partner.

These results highlight the critical importance of communication in driving the outcomes of collaborative recall in intimate couples. This central role for communication was predicted by transactive memory theory (Wegner, 1987; see also Barnier et al., 2018b) and confirmed in at least two prior studies of collaborative recall of older couples (Harris et al., 2011, 2018). If strategic, sensitive, and engaged communication – such as cuing, repetition, and rapid turn-taking – supports more effective collaboration, then hearing difficulties will invariably disrupt these processes of transactive memory or “distributed cognition” (Barnier et al., 2008; Harris et al., 2014b).

To illustrate the potential impact of hearing difficulties on collaborative recall, we briefly offer the case of Paul and Irene (not their real names), one of the long-married couples who participated in this study. At the time of testing, Paul was 87 years old, Irene was 77 years old, and they had been married for 57 years. Paul’s HHIE-S score was 30, representing severe self-reported hearing difficulties, whereas Irene’s score was 6, representing no hearing difficulties (although their verbal interactions transcribed below suggest otherwise for Irene). On the Mutual Friends task in Week 1, Paul recalled 9 names of mutual friends and acquaintances and Irene recalled 8 when they recalled individually with the experimenters. This gave them a total (pooled, nominal) score of 17 names at Week 1. When they worked together on this task 1 week later, they again recalled 17 names, experiencing no benefit from collaboration relative to remembering alone. This stands in contrast to the average collaborative benefit for all couples of nearly 17 names and the average collaborative benefit for couples without hearing difficulties of over 21 names (reported above).

When we looked closely at Paul and Irene’s conversation during the Mutual Friends task (and other tasks; see Barnier et al., 2018a), their failure of collaboration appeared to be due, at least in part, to difficulties in hearing. Based on these transcripts, individuals’ hearing difficulties appeared to lead to difficulties in tracking information offered by their spouse and difficulties in successfully cueing their spouse with useful memory prompts.

**Husband:** I can’t think of … Who lent you those books?
***Wife:*** *Pardon?*
**Husband:** Lent you the books and magazines. We’ve got to get them back to her.
***Wife:*** *I can’t hear you.*
**Husband:** She lent you the books and magazines.
***Wife:*** *Oh. Yeah.*
**Husband:** We’ve got to get them back to them. But I can’t think of their name.
***Wife:*** *No, I can’t either.*
**Husband:** I’m looking at a lot of people but I just can’t remember the names.

Finally, here is a segment of transcript from Paul and Irene’s conversation during a second task in which we asked couples to name European countries (reported in Barnier et al., 2018a):

**Husband:** Scotland. Ireland. England. France. Germany. Luxembourg. Norway. Holland. Um. Sweden.
***Wife:*** *I’m having terrible trouble hearing you.*
**Husband:** Sweden. Holland. I have said Luxembourg.
***Wife:*** *Ah Switzerland. Germany. Austria.*
**Husband:** Austria.
***Wife:*** *Latvia.*
**Husband:** Latvia.
***Wife:*** *Ukraine. Czechoslovakia.*
**Husband:** Spain. Italy.
***Wife:*** *France. Belgium. I don’t know whether we said that.*
**Husband:** Genoa. Russia.
***Wife:*** *Lithuania.*
**Husband:** Greenland.
***Wife:*** *Hmm?*
**Husband:** Greenland I said.
***Wife:*** *Speak up!*
**Husband:** Greenland! Turkey. No Turkey. That’s not part of Europe. Belgium.
***Wife:*** *Norway. Sweden. Finland.*
**Husband:** Scotland.
***Wife:*** *I don’t think we said Wales. Jutland.*
**Husband:** Hmm? Did you say Denmark?

Compare their collaboration to the following segment of transcript from a different couple’s conversation during the Mutual Friends task. In this case, neither spouse reported hearing difficulties, and their collaboration was characterized by cross-cuing with shared knowledge, effective coordination of recall, and turn-taking:

***Wife:*** … *and Glenda but I don’t know what her last name is*…
**Husband:** Glenda Warren.
***Wife:*** *Yeah. Julie Hooper.*
**Husband:** And Peter Hamilton.
***Wife:*** *Yeah. And Bridget and*…
**Husband:** Bridget and James Whitmore. Yes.
***Wife:*** *Okay where do we go now? Barry and Martha Gillis. Mirabelle and Graham Taylor. Jenny and Gary Tipper*…
**Husband:** You’re going through your Christmas list, ha ha.
***Wife:*** *Yes. Annabeth and Bill Boswell.*
**Husband:** Yeah. Katrina and Gomez Murray.

These examples underscore the importance of hearing and communication in successful memory collaboration.

There are several limitations to the current research, which means that these findings represent an exploratory first step in revealing a link between hearing loss and failures of memory scaffolding. Our sample was relatively small, especially when divided into hearing categories. We measured hearing difficulties *via* self-report of functional everyday impacts of hearing difficulties. Future research should include objective measures of hearing loss (e.g., audiometry, hearing performance in conversation). As is evident in the transcript above, self-report may not capture all cases or degrees of hearing loss. Whereas our measure of couple level hearing difficulties was relatively crude in simply adding spouses’ hearing scores, the impact of individual hearing loss within social systems such as long-married couples may be exponential rather than additive, emergent in combination with other factors, or buffered by still other factors (for further discussion of this problem of navigating from individual to couple levels of analysis, see Barnier et al., 2016, 2018b). We need to unpack the consequences of hearing difficulties for individuals as well as their most intimate partners (i.e., the third-party disability). Finally, we cannot establish causality in the current data, and future research should examine whether treating hearing loss *via* hearing aids or implants may lead to a recovery of collaborative benefits as well as whether other demographic or health variables play an important role in links between hearing and collaborative performance.

Despite these limitations, these findings have implications for more than just an individual who experiences everyday hearing difficulties. As members of distributed cognitive systems, we rely on one another to support and extend each other’s cognition (Barnier et al., 2008, 2014). For older adults in particular, the benefits of collaboration with a close family member or friend may protect (or compensate for) memory in the face of age- or disease-related decline (e.g., Kemper et al., 1995; Ross et al., 2004; Rauers et al., 2010; Hydén, 2011). However, when they are socially and cognitively cut off from the world that scaffolds them, hearing loss may place older adults at greater risk of cognitive decline. This possibility may help to explain significant, but still unexplained, links between hearing loss and increased dementia risk (e.g., Livingston et al., 2017) as well as the more recent and provocative links between marriage and reduced dementia risk (e.g., Sundström et al., 2016; Sommerlad et al., 2017). Interventions designed to support hearing, communication, and collaboration may prevent or delay cognitive decline and may even reduce dementia incidence in later life.

## Data Availability

The datasets generated for this study are available on request to the corresponding author.

## Ethics Statement

This study was carried out in accordance with the recommendations of the National Health and Medical Research Council’s National Statement on Ethical Conduct in Human Research (2007), the Australian Code for the Responsible Conduct of Research (2007), and the Macquarie University Code for the Responsible Conduct of Research. All subjects gave written informed consent in accordance with the Declaration of Helsinki. The protocol was approved by Macquarie University’s Human Research Ethics Committee and the Management Committee of the Australian Imaging, Biomarkers, and Lifestyle (AIBL) Study of Ageing.

## Author Contributions

AB, CH, TM, and GS developed and designed the study. GS facilitated access to the AIBL participants. TM and AB led the conduct of the study and data collection assisted by CH and two research assistants. AB and PS led the statistical analysis assisted by CH and TM. AB and PS drafted the manuscript with contributions from CH and TM. All authors contributed to manuscript revision led by AB and CH, and read and approved the submitted version.

## Conflict of Interest Statement

The authors declare that the research was conducted in the absence of any commercial or financial relationships that could be construed as a potential conflict of interest.
